# Functionalized chitosan–graphene magnetic nanocomposites enhance collagen synthesis in scleral fibroblasts

**DOI:** 10.1515/biol-2025-1316

**Published:** 2026-04-30

**Authors:** Xiaojie Huang, Zijian Wang, Min Zhang, Yingying Dai, Ruikang Tian

**Affiliations:** Zhejiang Industry and Trade Vocational College, Wenzhou, Zhejiang, China; Wenzhou Medical University, Wenzhou, Zhejiang, China

**Keywords:** myopia, scleral fibroblasts, magnetic stimulation

## Abstract

To investigate the effects of magnetic stimulation mediated by magnetic/graphene (MNP/G) composite nanomaterials on human scleral fibroblast (HFSF) proliferation and collagen synthesis, exploring its potential for myopia prevention. MNP/G nanomaterials were synthesized via covalent co-precipitation and introduced into HFSF cultures. Cells were co-cultured with 30 μg/mL MNP/G and exposed to static magnetic stimulation (20 mT intensity). Proliferation was assessed via CCK-8 assay, morphology was observed using inverted phase-contrast microscopy, and hydroxyproline content was measured to quantify collagen synthesis. Magnetic stimulation significantly enhanced HFSF proliferation compared to non-stimulated controls, with no observed cytotoxicity. Microscopic analysis revealed improved cell density and spindle-like morphology in stimulated groups. Hydroxyproline assays demonstrated a marked increase in collagen synthesis following magnetic stimulation, suggesting enhanced extracellular matrix production. Notably, the combined application of MNP/G nanomaterials and magnetic stimulation yielded the highest collagen content among all experimental conditions. Magnetic stimulation using MNP/G nanomaterials promotes HFSF proliferation and collagen production, potentially strengthening scleral biomechanical properties. This approach may counteract scleral remodeling in myopia progression, offering a novel strategy for non-invasive myopia intervention.

## 1 Introduction

Myopia has emerged as a global epidemic, with projections suggesting that by 2050, half of the world’s population will be affected. The increasing prevalence of myopia poses a substantial global health challenge [1]. Addressing myopia fundamentally requires prevention and control strategies rooted in its underlying causes and mechanisms. While the precise etiology of myopia remains unclear, one prominent hypothesis focuses on scleral remodeling [2]. It is believed that the axial elongation in myopia results from structural abnormalities in human scleral fibroblasts (HFSF), which cause abnormal scleral thickness and altered resistance to intraocular pressure. This, in turn, decreases scleral toughness and causes expansion deformation. To maintain optimal scleral toughness and stable performance, the repair capacity of scleral fibroblasts is essential, which is currently reflected in the proliferation of self generated cells to increase their quantity and/or thicken their collagen fibers [3], [4], [5], [6], [7]. Consequently, modulating the function of scleral fibroblasts has emerged as a pivotal strategy for myopia prevention and control. The reparative capacity of these fibroblasts is fundamental to maintaining the normal structural integrity and physiological toughness of the sclera. At this stage, such reparative potential is primarily characterized by the expansion of the cell population through proliferation or the thickening of the sclera via collagen fiber synthesis [8]. In cases of pathological myopia, the disordered arrangement of scleral collagen fibers can be explicitly visualized using transmission electron microscopy (TEM), while anomalous fluctuations in collagen content can be precisely quantified through advanced analytical techniques [9].

Magnetic nanoparticles (MNPs) have become a research hotspot in the field of nanomedicine in recent years due to their combination of nanoscale effects, controllable magnetic responsiveness, and modifiable surface properties [10]. Their magnetic targeting enables the precise delivery of biomaterials, while their superparamagnetism allows them to generate localized physical stimuli under an external magnetic field to modulate cellular functions. Furthermore, surface functionalization significantly enhances their biocompatibility, making them ideal vehicles for mediating non-invasive physical interventions and demonstrating significant application value in areas such as tissue repair and cellular regulation. Among these, iron oxide nanoparticles, as typical representatives of magnetic nanomaterials, exhibit unique advantages in fields such as drug targeting and gene transfection due to their excellent magnetic responsiveness and functional plasticity [11], [12], [13]. Magnetofection technology mediated by these particles facilitates highly efficient gene delivery, providing a novel approach for cellular function regulation. Graphene possesses exceptional electrical, chemical, optical, mechanical, and structural properties, positioning it as one of the most promising materials in the biomedical field [14]. As a biomaterial, functionalized graphene holds unique prospects for applications in regenerative medicine. Specifically, three-dimensional graphene foams – characterized by high porosity, robust mechanical support, and excellent biocompatibility – can regulate cellular adhesion, proliferation, and differentiation through surface modification [15]. Coupled with controllable biodegradability, these foams have become premium substrates for bone and neural tissue engineering scaffolds [16]. The application of graphene-based composites in bone tissue engineering is well-established, providing a valuable blueprint for their utilization in scleral tissue repair [17]. Furthermore, nitrogen-doping of graphene can further enhance its physicochemical properties, expanding the possibilities for advanced functional modifications [18].

As a natural cationic polysaccharide biomaterial, chitosan is widely utilized in nanomedicine due to the amino and hydroxyl groups on its molecular chain, which allow for multi-dimensional chemical modifications while providing excellent biocompatibility, biodegradability, and natural antimicrobial properties, rendering it an ideal vehicle for the functionalization of nanomaterials. In the field of drug delivery, customized nano-chitosan can achieve targeted and sustained drug release by modulating particle size and surface charge; in tissue engineering, macroporous chitosan scaffolds can mimic the three-dimensional microstructures of native tissues to provide structural support for cell growth and have been successfully applied in eyelid and cartilage repair research, while recent studies have also explored compositing chitosan with basil seed gum to construct hydrogel scaffolds – integrated with quercetin-loaded zein microspheres – to further enhance tissue-repair performance, offering novel perspectives for the architectural design of chitosan-based composite scaffolds; furthermore, in wound healing, chitosan-based composite nanofibers can accelerate tissue repair by modulating inflammatory responses and promoting angiogenesis, as exemplified by chitosan/poly (ethylene oxide)/CuFe_2_O_4_ nanofibers that exhibit dual synergistic effects in antimicrobial activity and tissue regeneration [19], [20], [21], [22]. Several research groups have utilized chitosan to fabricate chitosan-functionalized graphene, leveraging the bridging effect of chitosan to significantly enhance the biocompatibility and dispersibility of graphene in both cellular and animal models. This functionalization further imparts exceptional antibacterial properties to the composite material, demonstrating dual potential for antimicrobial therapy and tissue regeneration [23]. Flexible bactericidal graphene oxide-chitosan layers have been synthesized to achieve highly efficient antimicrobial activity while precisely regulating stem cell proliferation, providing a novel composite substrate for tissue engineering and repair [24].

From a historical perspective, the synergy between graphene and magnetic nanoparticles has evolved into a cornerstone of multifunctional nanomedicine. These nanocomposites have demonstrated exceptional performance in diverse clinical scenarios beyond simple drug delivery. For instance, zinc ferrite spinel-graphene has been successfully utilized in the magneto-photothermal therapy of cancer, where the combination of magnetic and optical properties significantly enhances tumor ablation efficiency through hyperthermia. Furthermore, graphene oxide-loaded magnetic nanoparticles integrated within 3D hydrogels have formed high-performance scaffolds for bone regeneration and tumor treatment, highlighting their ability to create bioactive environments that promote cell retention and structural repair. Chitosan–graphene magnetic nanocomposites (MNP/G) further consolidate the biocompatibility of chitosan, the structural integrity of graphene, and the magnetic actuation of nanoparticles. Due to their superior biocompatibility and versatile functional tunability, these composites exhibit unique potential for applications in tissue engineering and regenerative medicine [25], 26]. As a non-invasive physical intervention, static magnetic stimulation can achieve ocular tissue repair by modulating cellular proliferation and extracellular matrix synthesis without significant side effects. Furthermore, research regarding graphene-based antibacterial coatings has provided technical support for optimizing the antimicrobial performance of composite materials.

Current clinical strategies for myopia prevention and control, such as orthokeratology and low-concentration atropine, primarily focus on refractive correction or the inhibition of axial elongation [27]. However, there remains a significant scarcity of biomaterial-based intervention protocols specifically targeting scleral tissue repair. Furthermore, no reports currently exist regarding the application of magnetic stimulation technology combined with chitosan-graphene magnetic nanocomposites in the study of myopia development and progression. Although the influence of environmental factors on myopia has been extensively investigated, numerous paradoxes persist, presenting ongoing challenges for clinical practice in myopia prevention and control [28]. This study intends to use the previously reported edge functionalized ball milling method to prepare chitosan modified graphene, and further prepare graphene with superparamagnetism and biological properties through co precipitation method. Compatible Fe_3_O_4_ MNP/G composite nanomaterials were prepared by introducing MNP/G materials into HFSF cells. The magnetic field induced by the nanoparticles was used to generate a stimulating effect. The growth of HFSF cells following magnetic stimulation was observed, and the collagen content was analyzed to determine the impact on the toughness function of fibroblasts. A new prevention and correction method for myopia was proposed based on the mechanism of scleral remodeling to inhibit the occurrence and development of myopia.

## 2 Methods

### 2.1 Synthesis of MNP/G materials

#### 2.1.1 Preparation of functionalized graphene

We employed an environmentally friendly and convenient edge functionalized ball milling method, developed in our laboratory to ball mill graphite powder and chitosan, resulting in functionalized graphene in one step. According to previous literature, a weight ratio of chitosan to graphite within the range of 1:15–1:25 enables highly efficient functionalization of graphene while ensuring the biocompatibility of the resulting composite [29]. In preliminary studies, our research group established four weight ratio gradients (1:5, 1:10, 1:20, and 1:30) to evaluate the dispersibility and functionalization efficiency of graphene under different conditions. It was observed that at a 1:20 ratio, chitosan sufficiently encapsulates the edges of the graphene, preventing aggregation while preserving its exceptional physicochemical properties. Consequently, the mass ratio of graphite powder to chitosan was finalized at 1:20 for this study. First, graphite powder and chitosan were mixed in a mass ratio of 1:20 and added to the agate ball milling jar. The mixture was shaken to ensure even distribution and then ball-milled at a speed of 300–500 revolutions per minute for 8–12 h. Upon completion of the ball milling process, the agate jar was removed and rinsed with deionized water [30], 31]. A glass rod was used to stir the jar, fully dispersing the sample adhered to the jar walls and agate beads into deionized water, thereby obtaining a graphene dispersion. To remove small molecule impurities from graphene solution, the obtained sample was purified by dialysis, where small molecule solutes diffuse through the semi-permeable membrane into the surrounding liquid due to the concentration gradient. Firstly, cut a 10 cm long dialysis bag and boil it in deionized water for 20 min to activate it. Then, place the prepared graphene solution into a dialysis bag, seal both ends, and place it into a large beaker containing 3 L of deionized water. Change the water at the following intervals: 30 min, 1 h, 3 h, 6 h, 12 h, and 18 h for a total of six times to achieve impurity removal. Following this, proceed with further purification of the sample using the dissociation method. Transfer the graphene dispersion into a 50 mL centrifuge tube, then centrifuge at 1,000 rpm for 10 min. Finally, collect the supernatant and centrifuge the sample at 8,000 rpm for 10 min. Then, collect the sediment at the bottom, which contains the graphene needed.

#### 2.1.2 Preparation of MNP/G composite nanomaterials

The co-precipitation method is used to prepare MNP/G materials. This method generally involves mixing different substances in a liquid state, followed by the addition of suitable precipitants to form precursor precipitates, which can be dried to obtain corresponding solid particulate matter. The advantages of this method include mild reaction conditions, low cost, and simple process flow [32]. Additionally, the resulting product exhibits high purity and excellent dispersibility. First, the molar ratio of Fe^3+^/Fe^2+^ was strictly defined. Given that the theoretical molar ratio of Fe^3+^ to Fe^2+^ in the crystal structure of Fe_3_O_4_ is 2:1, this ratio ensures that the precursors react completely to form pure-phase Fe_3_O_4_ magnetic nanoparticles while preventing the formation of impurity iron oxides, such as Fe_2_O_3_. Furthermore, literature reports indicate that maintaining a Fe^3+^/Fe^2+^ molar ratio of 2:1 yields Fe_3_O_4_ nanoparticles with uniform particle size and excellent superparamagnetism, facilitating effective hybridization with graphene. Therefore, the molar ratio of ferric chloride hexahydrate (FeCl_3_ 6H_2_O) to ferrous chloride tetrahydrate (FeCl_2_ 4H_2_O) used in this experiment was precisely controlled at 2:1 [33], 34]. The specific operation steps are as follows: take 10 mL of the above-mentioned graphene dispersion and add it to a 250 mL three-neck round bottom flask. Then add 30 mg FeCl_3_ 6H_2_O, 380 mg FeCl_2_ 4H_2_O, and 30 mL deionized water, mix well by ultrasound, add nitrogen and oxygen removal. A continuous nitrogen purging method was employed, with the nitrogen flow rate regulated at 50 mL/min for a duration of 30 min. This step ensures the thorough removal of dissolved oxygen from the reaction system, thereby preventing the undesirable oxidation of Fe^2+^ ions [35]. The reaction is stirred at room temperature for 30 min before the temperature is raised to 70 °C, and stirring is continued for 8 h. Finally, the temperature is increased to 90 °C,and 2.5 mL of a 3 mol/L sodium hydroxide aqueous solution is added [36]. The reaction is allowed to proceed for an additional 3 h, resulting in the formation of MNP/G composite nanomaterials.

Precise control of the pH value is critical during the co-precipitation process. Prior to the dropwise addition of the aqueous sodium hydroxide (NaOH) solution, the initial pH of the reaction system is maintained between 2.5 and 3.0. As the NaOH is added slowly, the system’s pH is monitored in real-time until a final reaction endpoint of 10.0–10.5 is achieved. This specific pH range ensures the complete precipitation of Fe^3+^ and Fe^2+^ to form magnetite (Fe_3_O_4_). Concurrently, this prevents excessively high pH levels that could lead to the degradation of chitosan, thereby preserving the structural stability of the resulting composite materials [37].

The purification of MNP/G materials is achieved through magnetic separation, taking advantage of the magnetic properties of the target material. Transfer the material to a beaker, place a magnet on the surface of the beaker, and MNP/G can be separated from the solution. Wash repeatedly three times and then disperse again in 20 mL of deionized water. Place the MNP/G material dispersion in a glass culture dish, covered with a sealing film, and uniform small holes are made on the surface. The dish is frozen at −80 °C for 12 h in an ultra-low temperature freezer and subsequently freeze-dried for 24 h to obtain solid MNP/G.

### 2.2 Constructing a co-cultivation model of magnetic nanoparticles and HFSF

HFSF cells, obtained from the National Experimental Cell Resource Sharing Platform, will be cultured statically. After exchanging the medium, the cells will be designated as the first generation. They will be maintained in DMEM high glucose medium (DMEM/F12) supplemented with 20 % fetal bovine serum (FBS). After 2–3 medium changes, cells will be passaged once they reach 80–90 % confluence at the bottom of the culture flask. Following passaging, the cells will be cultured in DMEM/F12 medium supplemented with 10 % FBS and used for subsequent cell experiments [38].

#### 2.2.1 Determination of co-culture concentration using cell counting reagent CCK-8

The 2nd to 3rd generation HFSF cells will be used for the experiments. The cells are inoculated at a density of 5,000 cells per well into a 96-well plate and cultured in an incubator for 24 h. Observe the cell adhesion and normal growth status under an optical microscope. Then, use a pipette to aspirate the 96 well plate culture medium. MNP/G composite nanomaterials will be prepared in DMEM/F12 medium at concentrations of 5, 10, 15, 20, 25, 30, 35, 45, and 50 μg/mL, which have been disinfected by UV. The control group uses DMEM/F12 medium of equal volume and the plates will be incubated in the dark for 24 h. Following the instructions of the reagent kit, the medium will be replaced to conduct the CCK-8 assay. To minimize deviations in absorbance readings, care should be taken to avoid generating bubbles when adding 10 µL of CCK-8 reagent to each well. After adding the sample, wrap the 96 well plate with tin foil and incubate it in a 37 °C, 5 % CO_2_ incubator for 30 min. Then, read the value of absorbance (OD) at 450 nm on an enzyme-linked immunosorbent assay (ELISA) reader and create a bar chart according to the cell proliferation index = (experimental group A value – blank group A value)/(control group A value – blank group A value) × 100 %. The blank group consists of culture medium without cells. Each group will have five wells, and the experiment will be repeated three times [39], 40].

#### 2.2.2 Live cell staining, inverted phase contrast microscopy observation of cell morphology in magnetic stimulated HFSF

Literature reports indicate that static magnetic stimulation within a moderate intensity range of 20–50 mT can effectively promote fibroblast proliferation and collagen synthesis. Crucially, this specific range avoids inducing myofibroblast differentiation, thereby preventing excessive fibrosis and the abnormal elevation of tissue stiffness. In alignment with these safety thresholds, a stimulation intensity of 20 mT was selected for the present study [41]. Cell proliferation and survival rates were assessed using the CCK-8 assay under identical cell culture conditions and magnetic stimulation parameters. Concurrently, a staining solution was prepared at a ratio of Calcein AM: PBS buffer = 2 μL: 1 mL to perform light-avoiding staining on cells post-magnetic stimulation. Observe the growth status and quantity of cells with and without magnetic stimulation at different concentrations using an inverted phase contrast fluorescence microscope.

### 2.3 Determination of hydroxyproline standard curve

Strictly adhere to the instructions provided with the hydroxyproline assay kit to prepare and perform the hydroxyproline analysis [42]. (1) Dissolve a standard sample in double distilled water and dilute to 50 mL to prepare a standard stock solution of 100 μg/mL; (2) Dilute 100 μg/mL hydroxyproline standard stock solution with double distilled water to prepare standard solutions with the following concentrations (μg/mL): 20, 10, 5, 2.5, 1.25, 1, 0.5, and 0 μg/mL (blank), for subsequent use; (3) Transfer 0.25 mL of each standard solution into eight plastic centrifuge tubes (EP tubes) and add 0.25 mL of double-distilled water to a ninth EP tube as a blank control; (4) Add 0.05 mL of digestion solution to each of the 9 EP tubes, mix thoroughly, and incubate in a water bath at 37 °C for 3 h; (5) Add 0.5 mL of reagent 1 to each of the 9 EP tubes, mix well, and allow the tubes stand at room temperature for 10 min; (6) Add 0.5 mL of reagent 2 to each of the 9 EP tubes, mix thoroughly, and allow the tubes to stand at room temperature for 5 min; (7) Add 1.0 mL of reagent three to each of the nine EP tubes, mix thoroughly, and incubate in a water bath at 60 °C for 15 min; (8) After cooling the 9 EP tubes under running water, set the UV spectrophotometer to 550 nm, use double-distilled water as the blank to zero the instrument, and measure the absorbance of each tube using a 1 cm optical path [43].

### 2.4 Determination of hydroxyproline content

#### 2.4.1 Cell experiment seed plate

(1) HFSF cells were cultured in an incubator as described previously. Once cell adhesion and normal growth were observed under an optical microscope, DMEM/F12 medium was used to culture the cells until they reached 80–90 % confluence. Cells were then prepared for seeding experiments. Two 48-well plates were used, with 10,000 cells per well, eight wells per plate, and four groups per vertical column. The plates were incubated at 37 °C for 24 h to allow cell adhesion. The groups were labeled as follows: Group 1: Stimulus−/Material+; Group 2: Stimulus−/Material−; Group 3: Stimulus+/Material−; Group 4: Stimulus+/Material+. (2) After cell adhesion, the magnetic nanoparticle materials were disinfected and diluted according to the experimental group specifications. The cells were then cultured for an additional 24 h at 37 °C in the incubator. (3) For the groups requiring magnetic stimulation, the plates were placed on a magnetic stimulator for 1 h and then incubated for another 24 h. The parameters of the stimulator were set to static stimulation with an intensity of 20 mT. (4) Upon completion of the cultivation, the cells were collected for hydroxyproline content measurement using a spectrophotometer. Briefly, the following steps were performed. The cultured cells were digested with trypsin for 3 min. After digestion, the same volume of complete culture medium was added, and the cells were transferred into four 1.5 mL EP tubes according to the experimental groupings. The cells were then disrupted using a cell disruptor, followed by centrifugation at 1,500 rpm for 5 min. The supernatant was discarded.

Hydroxyproline content was determined using the hydroxyproline assay kit. The following steps were performed for each tube (experimental, blank, and standard): (a) Add 5 mL of double distilled water to each EP tube, for the experimental group, the blank tube, and the standard tube. The blank tube contained only double-distilled water, while the standard tube contained 5 mL of a 5 μg/mL standard solution. (b) The cells in the experimental tube were disrupted using an ultrasonic disruptor (time: 20 s; power: 30 %). (c) Add 0.05 mL of digestion solution to the experimental tube, blank tube, and standard tube respectively, mixed well, and water bath at 37 °C for 3 h. (d) Add 0.5 mL of reagent 1 from the kit to the experimental tube, blank tube, and standard tube respectively, mixed well, and allowed to stand at room temperature for 10 min. (e) Add 0.5 mL of reagent 2 from the reagent kit to the experimental tube, blank tube, and standard tube respectively, mixed well, and allowed to stand at room temperature for 5 min. (f) Add 1.0 mL of reagent 3 from the reagent kit to the experimental tube, blank tube, and standard tube respectively, and water bath at 60 °C for 15 min. (g) After cooling to room temperature with running water, the tubes were centrifuged at 3,500 rpm for 10 min. (h) Take the supernatant at 550 nm, 1 cm optical path, adjust to zero with double distilled water, and measure the absorbance (OD) of each tube. (i) The hydroxyproline concentration in the experimental tubes was calculated based on the regression equation derived from the standard curve in Section 2.3. This value was subsequently used to determine the total collagen synthesis of the cells. Each experimental group was performed in triplicate, and the mean value of the three independent replicates was recorded as the final result.

## 3 Results and discussion

The synthesis of hydrophilic functionalized graphene via the ball milling of chitosan and graphite powder was previously developed and characterized by our research group. During the mechanochemical process, chitosan is initially functionalized at the edges of graphite sheets through solid-phase mechanochemical reactions, and then continuously added inward to expand the space between the layers of graphite sheets. The force of ball milling promotes the peeling of nano graphene sheets. Literature reports indicate that the D-band (approximately 1,350 cm^−1^) and G-band (approximately 1,580 cm^−1^) in Raman spectra serve as characteristic identifiers for graphene, where the D-band corresponds to edge defects and the G-band to the stretching vibrations of sp^2^ hybridized carbon atoms. Additionally, the 2D-band (approximately 2,700 cm^−1^) is utilized to evaluate the layer thickness of graphene. Previous studies employing the same ball-milling method to prepare chitosan-functionalized graphene have detected distinct D, G, and 2D bands, confirming successful exfoliation. In the present study, the thin-layered morphology of graphene observed via TEM is consistent with these reported findings, indirectly validating the presence of graphene in the samples. Furthermore, the attachment of magnetic nanoparticles observed under TEM, combined with the characteristic infrared (FT-IR) absorption peaks, provides further evidence for the successful synthesis of the MNP/G nanocomposites [44].

**Figure 1:**
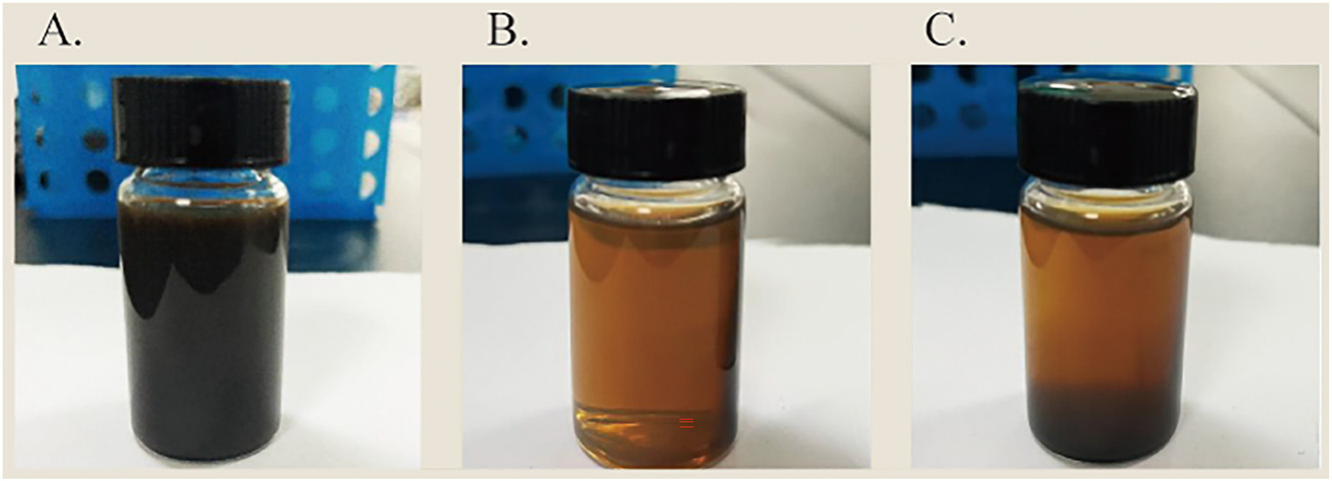
Magnetic responsiveness of the MNP/G nanocomposites. (A) Dispersed MNP/G solution. (B) MNP/G under magnet adsorption for 20 min. (C) MNP/G after removing the magnet for 30 s.

The highly dispersed superparamagnetic magnetic nanoparticle composite, prepared by adding magnetic Fe_3_O_4_ nanoparticles to functionalized graphene, may affect cell growth through conjugation with external magnetic fields. The prepared magnetic nanoparticle composite can be dissolved in deionized water to obtain a highly dispersed aqueous solution, as shown in Figure 1.

When an external magnetic field is applied, a strong magnetic force of MNP/G can be seen along the direction of magnetic field, as shown in Figure 1, indicating that MNP/G can be easily separated from the solution [45].

By observing Figure 1, it is evident that the MNP/G solution exhibits extremely strong magnetism [46].

**Figure 2:**
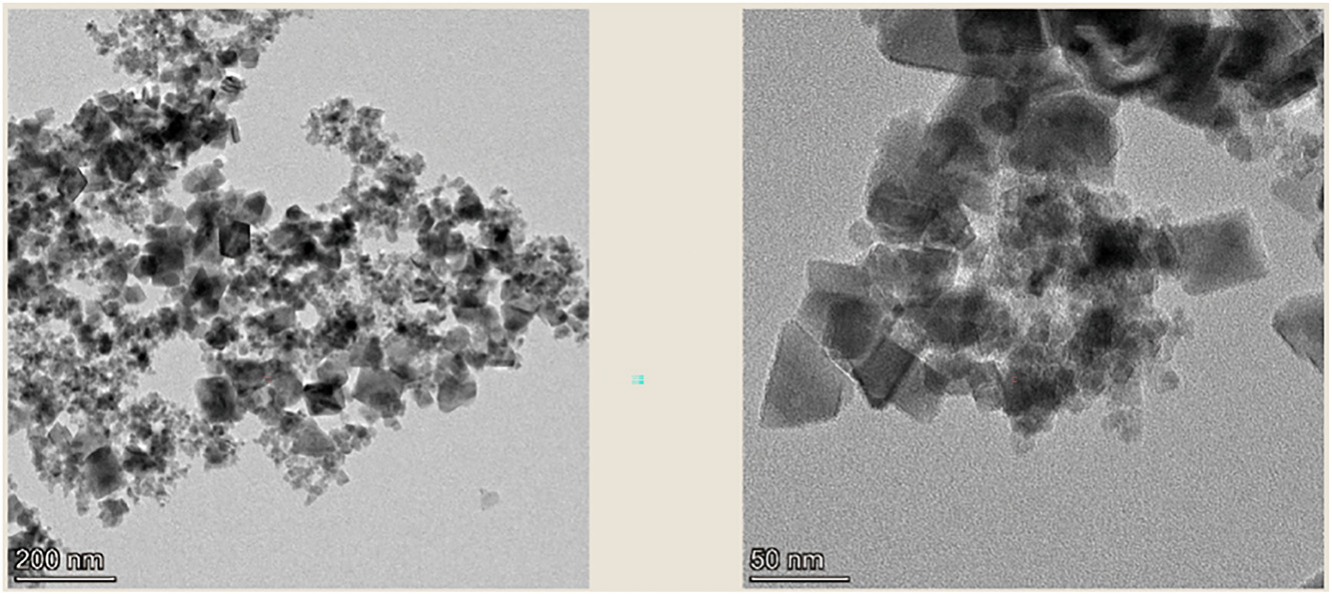
TEM of MNP/G composite nanomaterials.

**Figure 3:**
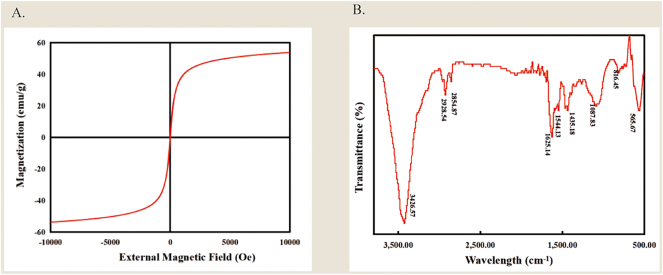
Magnetic and chemical characterization of the MNP/G nanocomposites. (A) Hysteresis curve of MNP/G composite nanomaterials. (B) FT-IR infrared spectrum of MNP/G composite nanomaterials.

The morphology of MNP/G composite nanomaterials was characterized by TEM under different magnifications. Figure 2 shows that the MNP/G composite nanomaterials are very uniform and well-dispersed, with magnetic nanoparticles visible on the graphene layers. The magnetic properties of MNP/G composite nanomaterials were determined and analyzed using a superconducting quantum interference device at room temperature. The results, as shown in Figure 3A, showed no hysteresis or remanent magnetism within the magnetic field strengths of ±1 × 10^4^ Oe, but rather presented as a curve passing through the coordinate origin, indicating good paramagnetism. The saturation magnetization strength of MNP/G was approximately 55 emu/g.

Hysteresis loop measurements were performed in triplicate for each sample (*n* = 3), with data expressed as mean ± standard deviation. For Fourier-transform infrared (FT-IR) spectroscopy, samples were prepared using the KBr pellet method and scanned over a range of 400–4,000 cm^−1^. Each sample was scanned three times to obtain the representative average spectrum.

The successful preparation of MNP/G composite nanomaterials is confirmed by the physical absorption peaks observed in infrared spectroscopy, as shown in Figure 3B. The absorption peak at 565 cm^−1^ in the curve is the Fe–O vibration peak of iron oxide in MNP/G composite nanomaterials. The curve at 1,625 cm^−1^ corresponds to the C=O peak of graphene in MNP/G composite nanomaterials. The absorption peak here is the stretching vibration of bonds in carboxylic acids. The peak at 3,426 cm^−1^ is likely a multiple absorption peak formed by hydrogen bonding of chitosan in magnetic nanoparticles, including the – OH stretching vibration absorption peak and the – NH_2_ stretching vibration absorption peak. The simultaneous appearance of characteristic absorption peaks at 565 cm^−1^, 1,625 cm^−1^, and 3,426 cm^−1^ confirms the co-existence of Fe_3_O_4_, graphene, and chitosan within the sample, thereby verifying the successful synthesis of the MNP/G nanocomposites. These results are consistent with the FT-IR spectral features reported in previous studies for chitosan-graphene-Fe_3_O_4_ composites, demonstrating the successful synthesis of MNP/G composite nanomaterials.

**Figure 4:**
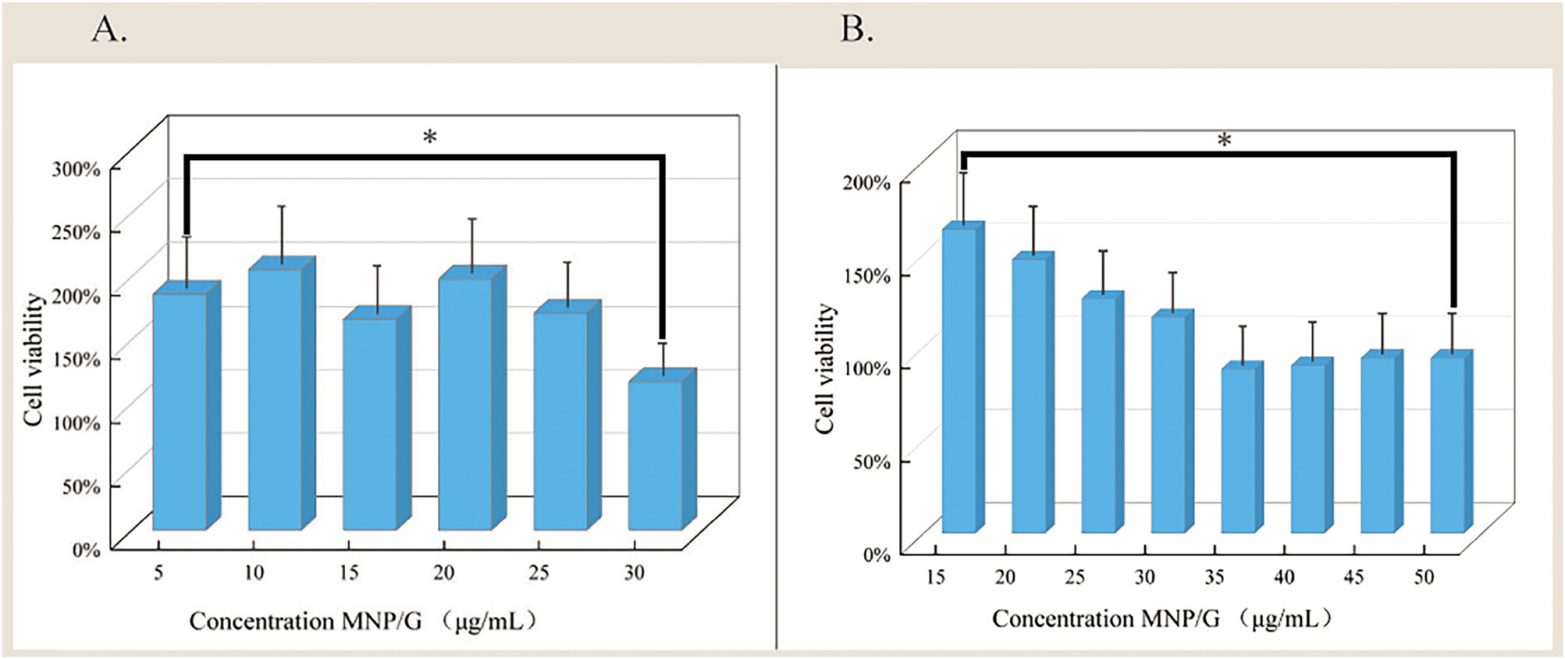
Effect of different concentrations of MNP/G composite nanomaterials on the proliferation of HFSF cells. Cells were treated with indicated concentrations for 24 h. Cell viability was assessed by CCK-8 assay. Data are presented as mean ± SD (*n* = 5 wells per group, from three independent experiments). Statistical significance was determined by one-way ANOVA followed by Dunnett’s post-hoc test for comparison with the control group. *P < 0.05, **P < 0.01 compared to control.

The CCK-8 method was used to determine the effect of different concentrations of MNP/G composite nanomaterials on HFSF cell proliferation. For each experimental group, five replicate wells were established, and the experiments were performed in triplicate (*n* = 3). Data are expressed as the mean ± standard deviation. Statistical analysis was conducted using one-way analysis of variance (ANOVA) followed by Dunnett’s post-hoc test. Compared to the control group, the cell viability of the 30 μg/mL concentration group showed no significant difference (*P* > 0.05). Similarly, the 50 μg/ml concentration group yielded a *P*-value > 0.05, indicating no significant cytotoxicity. At concentrations below 30 μg/mL, cell growth increased as the concentration decreased, while concentrations above 30 μg/mL caused slight inhibition. A concentration of 30 μg/mL approximates the natural growth state of normal HFSF cells. This study aims to assess the effect of magnetic stimulation on cell growth. Therefore, in order to eliminate the influence of the material itself on cell growth, a concentration of 30 μg/mL MNP/G composite nanomaterials was selected for co-culture in subsequent experiments (Figure 4).

Consequently, a concentration of 30 μg/mL of MNP/G composite nanomaterials was selected as the optimal dose for subsequent co-culture experiments. Notably, while previous studies have reported that high concentrations of graphene oxide can disrupt cell cycle progression and induce apoptosis in fibroblasts, the present study demonstrates that chitosan-functionalized graphene magnetic nanocomposites at 30 μg/mL do not impair HFSF viability. This observation aligns with existing literature, which identifies surface functionalization and dose control as critical determinants of the cytotoxicity of graphene-based materials. Chitosan, as a functionalization carrier, plays a pivotal role: the amino groups on its molecular chains can neutralize the negative charge on the graphene surface, thereby reducing graphene-induced cell membrane damage and enhancing biocompatibility. Moreover, the concentration of 30 μg/mL falls within the non-cytotoxic range for graphene-based materials, thus avoiding cell cycle disruption and apoptosis [47], 48]. These findings further underscore the rationale behind the surface functionalization design and concentration selection in this study.

Finally, a 30 μg/mL MNP/G composite nanomaterial solution was selected as the co-culture concentration.

**Figure 5:**
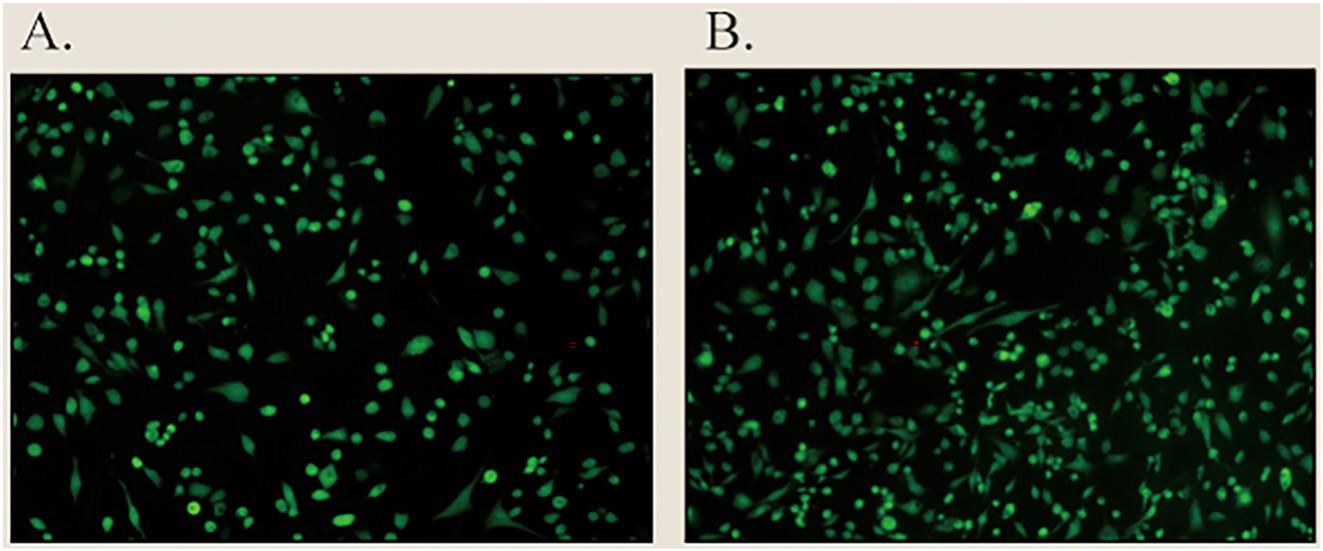
Representative images of HFSF cell growth before and after magnetic stimulation. Cells were co-cultured with MNP/G composite nanomaterials and stained with Calcein AM. (A) Without magnetic stimulation. (B) With magnetic stimulation.

The effect of magnetic stimulation on HFSF growth was assessed by staining live cells, with live cells represented in green in Figure 5. Live cell staining was performed using the Calcein-AM assay. Under fluorescence microscopy, the excitation and emission wavelengths were set at 488 nm and 520 nm, respectively. For each sample, five different fields of view were randomly selected for imaging and cell counting (*n* = 5). Statistical analysis was conducted using the independent samples *t*-test. The difference in cell number between the magnetic stimulation group and the non-stimulated control group yielded a *P*-value < 0.01, indicating a highly significant statistical difference. Observation under an inverted phase contrast microscope revealed that when a 30 μg/mL MNP/G composite nanomaterial solution was used for co-culture, a significant increase in cell number was observed under magnetic stimulation, while the cell morphology remained largely unchanged. Panel a shows cells without magnetic stimulation, while panel b shows cells subjected to magnetic stimulation.

Literature reports suggest that magnetic stimulation can regulate cell proliferation via mechanotransduction, an effect that may be influenced by the internalization status of MNP/G materials. Although this study did not directly assess the entry of MNP/G into HFSFs, prior research has demonstrated that graphene-based magnetic nanomaterials at a concentration of 30 μg/mL can enter fibroblasts through endocytosis. Furthermore, functionalization with chitosan has been shown to facilitate cellular internalization. Concurrently, magnetic stimulation may modulate cell proliferation by transmitting mechanical signals through internalized MNP/G materials or by acting directly on mechanoreceptors on the cell membrane, with these two pathways potentially working in synergy. In our experiments, the cell proliferation observed in the group subjected to both magnetic stimulation and materials (Tube 4) was superior to that in the group receiving magnetic stimulation alone (Tube 3). This indicates a synergistic effect between the MNP/G materials and magnetic stimulation. We hypothesize that the underlying mechanism involves the internalization of MNP/G, which, under magnetic stimulation, transmits stronger mechanical signals intracellularly to further promote cell proliferation. This hypothesis aligns with existing mechanisms described in studies on the regulation of cellular function via magnetic stimulation combined with magnetic nanomaterials.

**Figure 6:**
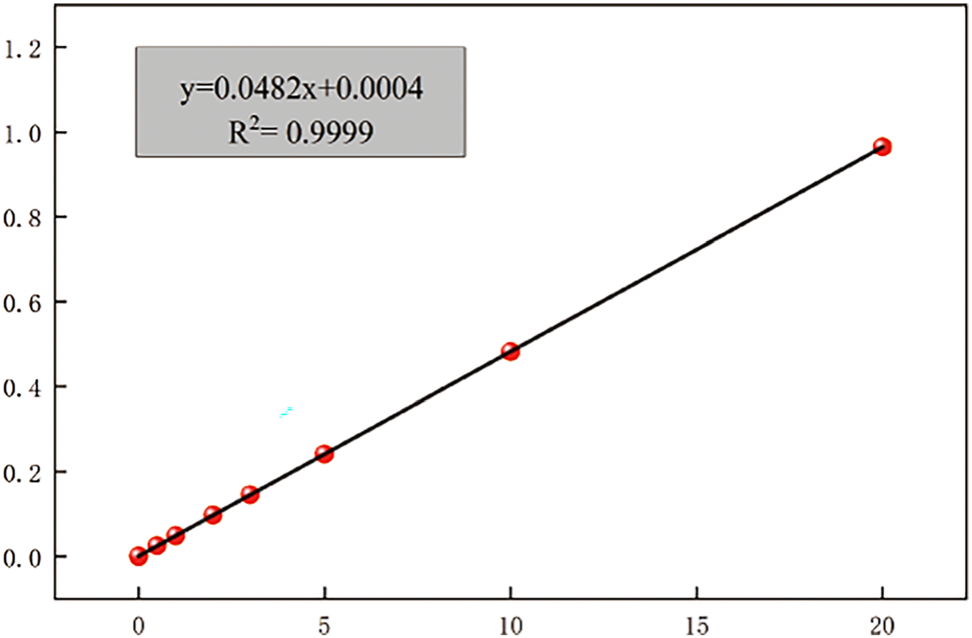
Standard fitting curve of hydroxyproline.

Scleral fibroblasts and extracellular matrix are the main components of the human sclera. The sclera’s strong toughness is crucial for maintaining the eyeball’s intrinsic shape. It has been suggested that the sclera determines the direction of eye development, whether towards emmetropia or pathological myopia. Scleral collagen is the main component of the sclera. While hydroxyproline is an indirect marker of collagen, a substantial body of research has established it as a reliable quantitative index for assessing collagen synthesis. Furthermore, its detection levels demonstrate strong correlation with both COL1A1 gene expression and collagen protein content as measured by Western blot analysis. Table 1 and Figure 6 show the absorption values of hydroxyproline at various concentrations and the standard curve of hydroxyproline obtained by the hydroxyproline kit.

**Table 1:** Absorption values of hydroxyproline at various concentrations.

Standard concentration (ug/mL)	Measured OD value	Absolute OD value
0	0.0768	0
0.5	0.0965	0.0197
1	0.1271	0.0503
1.25	0.1367	0.0599
2.5	0.2031	0.1263
5	0.3207	0.2439
10	0.5554	0.4786
20	1.0425	0.9657

For each concentration in the hydroxyproline assay, three replicate wells were established (*n* = 3), and the absorbance values are expressed as the mean ± standard deviation. A standard curve was constructed using linear regression analysis. The resulting regression equation, *y* = 0.0483 + 0.0012, with a coefficient of determination *R*’ = 0.9995, demonstrates excellent linearity. This validates the curve’s reliability for the subsequent calculation of hydroxyproline content in the experimental samples.

The standard curve was plotted using Origin 9.0 software. Linear regression analysis yielded acoefficient of determination *R*’ = 0.9995 with *P* < 0.001, indicating a highly significantstatistical relationship. These results confirm that the standard curve is robust and suitable for the precise determination of hydroxyproline content in subsequent experimental samples.

The MNP/G synthesized via the covalent precipitation method exhibits robust paramagnetism, which may affect the growth of scleral fibroblast cells under an external magnetic field. Based on the purpose of this study, the concentration absorption values of hydroxyproline in scleral fibroblast cells before and after magnetic stimulation were detected by a UV spectrophotometer, and changes in hydroxyproline content in each group were calculated by referencing the standard curve, as shown in Table 2.

**Table 2:** OD value of hydroxyproline and hydroxyproline content in each group.

Group	Measure OD value	Absolute OD value	Concentration (ug/mL)
Pipe No.1	0.067	0.0033	0.06
Pipe No.2	0.0664	0.0027	0.048
Pipe No.3	0.0695	0.0058	0.112
Pipe No.4	0.0747	0.011	0.22
Blank tube	0.0637	0	–
Sandard pipe	0.4975	0.4338	5

Tube 1: Stimulus−/Material+; Tube 2: Stimulus−/Material−; Tube 3: Stimulus+/Material−, Tube 4: Stimulus+/Material+.

As shown by the experimental data in Table 2, the hydroxyproline content in HFSFs significantly increased following magnetic stimulation, indicating that magnetic stimulation promotes collagen synthesis in these cells. Previous studies have reported that the molecular mechanisms underlying magnetic stimulation-induced collagen synthesis in fibroblasts involve several key signaling pathways, primarily the TGF-β/Smad, integrin-FAK, and MAPK pathways. Specifically, the TGF-β/Smad pathway serves as a core regulator; magnetic stimulation can upregulate TGF-β1 expression, leading to the phosphorylation of Smad2/3 and subsequent activation of collagen genes such as COL1A1. Additionally, the integrin-FAK pathway facilitates mechanotransduction, where magnetic stimulation may activate integrin receptors to promote FAK phosphorylation, thereby regulating cell proliferation and collagen synthesis. The MAPK signaling pathway, via ERK1/2 phosphorylation, also plays a critical role in modulating fibroblast proliferation and collagen secretion. The enhanced collagen synthesis observed in our study aligns with these reported mechanisms. Although direct protein expression analysis of these signaling pathways was not conducted in the current study, it is reasonable to hypothesize, based on the established literature, that the promotion of collagen synthesis in HFSFs by magnetic stimulation in our system is associated with the activation of the TGF-β/Smad, integrin-FAK, and MAPK pathways [49], [50], [51]. This provides a focused direction for our future investigations into the specific molecular mechanisms involved.

Differentiation of fibroblasts into a myofibroblast phenotype can directly impair normal physiological tissue function, Literature defines myofibroblasts as a specialized differentiated phenotype characterized primarily by the expression of α-smooth muscle actin (α-SMA), a process regulated by the TGF-β/Smad signaling pathway [52]. However, existing studies indicate that moderate-intensity static magnetic stimulation (20–50 mT) promotes fibroblast proliferation and collagen synthesis without significantly inducing differentiation into myofibroblasts, thereby avoiding excessive fibrosis. Consequently, this study employed a static magnetic stimulation intensity of 20 mT, consistent with the parameters cited in the literature. We hypothesize that this intensity will not induce HFSF differentiation into a myofibroblast phenotype, allowing for the enhancement of collagen synthesis and scleral toughness while maintaining the normal physiological function of scleral tissue. This hypothesis requires further verification in subsequent experiments.

Long-term exposure experiments are instrumental in evaluating the longitudinal impacts of MNP/G materials and magnetic stimulation on HFSFs, specifically regarding the stability of cell proliferation, the persistence of collagen synthesis, and the chronic biocompatibility of the materials. Prior research has indicated that the regulatory effects of magnetic stimulation combined with graphene-based magnetic nanomaterials on fibroblasts are sustained; following long-term exposure (up to 72 h), both cell proliferation and collagen production were maintained at stable levels without manifesting significant long-term toxicity. Extrapolating from these findings, we hypothesize that the MNP/G materials and magnetic stimulation in our study will exhibit similar long-term stability and safety, effectively promoting HFSF growth and extracellular matrix production over extended periods. While the current study focuses on the initial response (24–48 h) to establish proof-of-concept, we intend to incorporate extended exposure protocols in our future *in vivo* and *in vitro* studies to further validate this hypothesis, thereby providing a more robust evidentiary basis for the clinical translation of MNP/G materials in myopia prevention and control.

Furthermore, chitosan-based hydrogels hold significant value in tissue regeneration. Similar to the pH-sensitive gelation of chitosan hydrogels utilized in myocardial regeneration, the chitosan-functionalized graphene system in this study highlights the critical role of chitosan under physiologically relevant conditions. The pH-responsive properties of chitosan allow it to form biomimetic gel-like structures at a physiological pH of 7.35–7.45, a mechanism consistent with that observed in cardiac repair [53]. Additionally, chitosan facilitates cellular attachment and retention by binding to cell surface receptors via its amino groups, thereby promoting the synthesis of the extracellular matrix. In the present experiment, the chitosan-functionalized graphene significantly enhances the biocompatibility of the MNP/G materials, stimulating the proliferation of HFSFs and collagen synthesis through a mechanism analogous to that of chitosan hydrogels in myocardial regeneration. These findings further substantiate the pivotal role of chitosan in tissue engineering and provide robust literary support for the application of MNP/G materials in scleral tissue repair.

## 4 Conclusions

The sclera is the outermost layer of the eyeball, maintains its normal shape due to its toughness. The toughness of the sclera relies on its extracellular matrix and collagen fibers. Scleral fibroblasts, an important component of the sclera tissue, secrete collagen fibers and constitute the primary extracellular matrix. Collagen, the main component, contains a higher concentration of hydroxyproline compared to other proteins. This study found that magnetic stimulation promotes the growth of HFSF within a safe concentration range, with no significant difference in cell morphology compared to normal growth. Measurement of collagen content showed that magnetic stimulation promotes collagen secretion in scleral fibroblasts, suggesting an increase in scleral toughness. Research has shown that the sclera of highly myopic eyes exhibits disordered arrangement of collagen fiber with varying thickness. If the toughness of scleral fibroblasts increases, we can study the mechanism of the occurrence and development of axial myopia. For example, we could investigate whether magnetic stimulation increases fibroblast viscoelasticity, enhances scleral elasticity, and delays myopia progression. This study is based on the mechanism of myopia and scleral remodeling, innovatively uses magnetic stimulation technology to HFSF cells, activating transmembrane pathways and triggering a series of cell damage repair mechanisms, achieving self regeneration and repair of scleral injury sites. It is expected to achieve the goal of “mirror free” myopia prevention and correction, and provide new methods for myopia prevention and control technology.
